# Factors Influencing Patient Presentation and Transfer to Hospital Rates During Mass-Gathering Stadium Events: A Scoping Review

**DOI:** 10.1017/S1049023X25000287

**Published:** 2025-04

**Authors:** Nazneen Sultana, Julia Crilly, Robert S. Ware, Jamie Ranse

**Affiliations:** 1. School of Nursing and Midwifery, Griffith University, Gold Coast, Queensland, Australia; 2. Griffith Biostatistics Unit, Griffith Health, Griffith University, Queensland, Australia; 3. Department of Emergency Medicine, Gold Coast Health, Gold Coast, Queensland, Australia; 4. Centre for Mental Health, Griffith University, Queensland, Australia; 5. School of Medicine and Dentistry, Griffith University, Queensland, Australia

**Keywords:** arena, crowding, Emergency Medical Service, events, mass gathering, scoping review, stadium

## Abstract

**Introduction:**

Mass-gathering events (MGEs) such as sporting competitions and music festivals that take place in stadiums and arenas pose challenges to health care delivery that can differ from other types of MGEs. This scoping review aimed to describe factors that influence patient presentations to in-event health services, ambulance services, and emergency departments (EDs) from stadium and arena MGEs.

**Method:**

This scoping review followed the Preferred Reporting Items of Systematic Reviews and Meta-Analysis for Scoping Reviews (PRISMA-ScR) checklist and blended both Arksey and O’Malley methodology and the Joanna Briggs Institute’s (JBI’s) approach. Four databases (CINAHL, Embase, PubMed, and Scopus) were searched using keywords and terms about “mass gatherings,” “stadium” or “arena,” and “in-event health services.” In this review, the population pertains to the spectators who seek in-event health services, the concept was MGEs, and the context was stadiums and/or arenas.

**Results:**

Twenty-two articles were included in the review, most of which focused on sporting events (n = 18; 81.8%) and music concerts (n = 3; 13.6%). The reported patient presentation rate (PPR) ranged between one and 24 per 10,000 spectators; the median PPR was 3.8 per 10,000. The transfer to hospital rate (TTHR) varied from zero to four per 10,000 spectators, and the median TTHR was 0.35 per 10,000. Key factors reported for PPR and TTHR include event, venue, and health support characteristics.

**Conclusions:**

There is a complexity of health care delivery amid MGEs, stressing the need for uniform measurement and continued research to enhance predictive accuracy and advance health care services in these contexts. This review extends the current MGE domains (biomedical, psychosocial, and environmental) to encompass specific stadium/arena event characteristics that may have an impact on PPR and TTHR.

## Introduction

A mass-gathering event (MGE) is a planned or spontaneous event where the number of attendees may overwhelm the planning and response resources of the community, state, or nation hosting the event.^1^ There are various types of MGEs, including concerts, sporting events, religious celebrations, street fairs, parades, and political rallies. Each event has a distinct risk profile, with the nature of the event playing a role in determining the associated risks.^2^ For instance, stadium events are considered bounded, while marathons are unbounded, and this distinction affects the risks of injury or illness. Moreover, attending concerts and sporting events poses certain hazards that can include the possibility of recreational drug and alcohol consumption.^3^ Religious gatherings such as Hajj pilgrimage often comprise a majority of older people, which introduces additional considerations for health preparedness.^4^

Mass-gathering event health care involves providing organized public health and emergency medical care to individuals who gather at a specific location for a defined period of time.^5–7^ These MGEs are complex and can present unique challenges to attendees’ health and well-being. During such MGEs, some participants may require health care for injuries or illnesses, and it is essential to have an in-event health service to limit the impact of MGEs on ambulance and emergency department (ED) services.^8^ By recording the number of people seeking medical attention, organizers, health care providers, and emergency response teams can better understand the scope and nature of health-related challenges that arise during an MGE. Such information can be used to inform improvements in health care provision.

**Figure 1.**
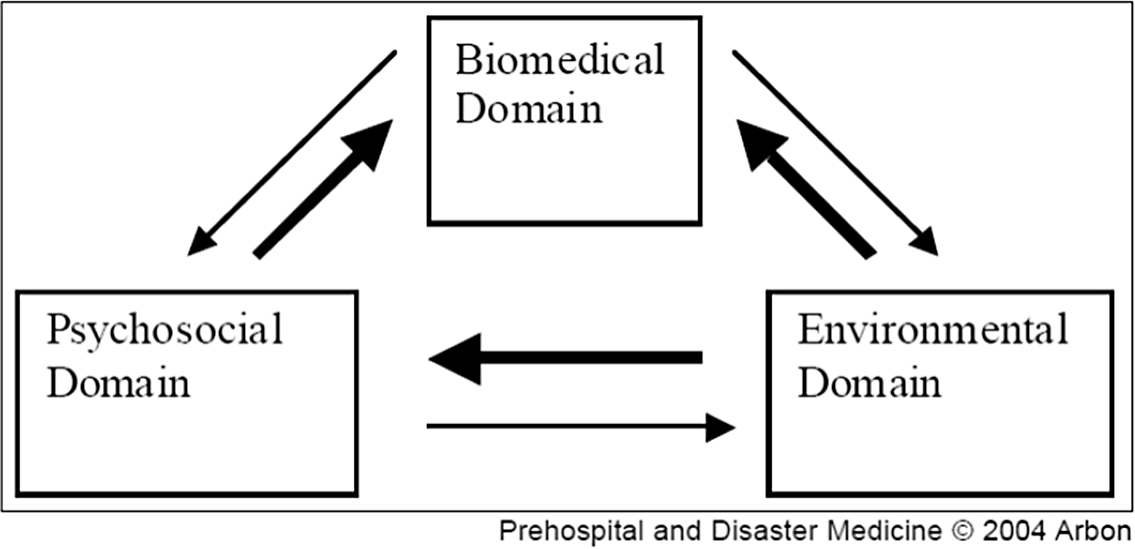
The Relationship Model of Domains for MGE.^2^ Note: Used with permission.

The patient presentation rate (PPR) is one metric to measure health care usage at an MGE.^4^ The transfer to hospital rate (TTHR), defined as the rate at which individuals attending an MGE require transportation to a hospital for further medical attention, is another metric.^9^ Particular factors are known to influence PPR and TTHR from MGEs.^10–12^ In 2004, a fundamental conceptual model for MGEs was proposed to understand and identify three interconnected domains: biomedical, environmental, and psychosocial^2^ (Figure 1). The original model has evolved with the inclusion of additional domains of the event environment, command, control, communication, public health, health promotion, and legacy.^3^ However, due to nuances between MGE types, the determinants specifically related to stadium/arena-based MGEs need to be considered to inform further research. The research question guiding this scoping review was: What factors influence patient (spectators) presentations to in-event health services, ambulance services, and EDs from stadium and arena MGEs?

## Methods

### Design

The scoping review followed the Preferred Reporting Items of Systematic Reviews and Meta-Analysis for Scoping Reviews (PRISMA-ScR) checklist^13^ and elements of the Joanna Briggs Institute (JBI; Adelaide, Australia) methodology,^14^ which includes an outline of the framework proposed by Arksey and O’Malley.^15^ Arskey and O’Malley’s framework consists of six stages. These stages include (1) identifying the research question; (2) identifying relevant studies; (3) study selection; (4) charting the data; (5) collating, summarizing, and reporting the results; and (6) consultation (optional).

### Search Strategy

With the research question (Stage 1) articulated above, Arskey and O’Mally’s framework^15^ was used to identify relevant studies (Stage 2). Databases were searched in April 2023 (Supplementary Material; available online only). There was no start date for the search, therefore all papers published through April 2023 were included in this search strategy. A thorough search of four different databases was undertaken to gather relevant articles. The databases searched were CINAHL Complete (EBSCO Information Services; Ipswich, Massachusetts USA); Embase (Elsevier; Amsterdam, the Netherlands); PubMed (National Center for Biotechnology Information, National Institutes of Health; Bethesda, Maryland USA); and Scopus (Elsevier; Amsterdam, the Netherlands). To capture pertinent articles, Medical Subject Headings (MeSH) terms and keywords that were specific to MGEs, different types of stadium and arena events, and in-event health services were used. The search strategy considered the Population, Concept, and Context method recommended by the JBI for scoping reviews.^14^ In this review, the population pertained to the spectators who seek in-event health services, the concept was MGE, and the context was stadium and/or arenas. A comprehensive list of MeSH terms and keywords is shown in Table 1. To maximize the search results, authors combined terms and keywords in the columns using the OR search strategy, while terms and keywords in the rows were combined using AND combinations.

**Table 1.** MeSH Terms and Keywords

	Mass Gatherings	Stadium/Arena Types	In-Event Health Services
MeSH Terms	Mass Gathering Crowding Anniversaries and Special Events	Track and Field	First Aid Ambulances Emergency Medical Services Health Personnel Emergency Treatment Emergency Medical Technicians Nurses Physicians Medical Staff Sports Medicine
Keywords	Large Event Major Event Mass Event Event Planning	Stadium Arena Ground Field Colosseum	Doctor Health Care Patient Presentations Transport to Hospital Paramedic Medical Care

Stage 3 of Arskey and O’Mally’s framework^15^ “study selection” included the identification of inclusion and exclusion criteria necessary to establish the review’s boundaries and to identify the studies that aligned with the review question. The inclusion and exclusion criteria of this scoping review are presented in Table 2.

**Table 2.** Inclusion and Exclusion Criteria

Inclusion Criteria	Exclusion Criteria
Reporting on real-world mass-gathering events in stadium or arena Published in English Articles that discussed influencing factors such as weather conditions, crowd demographics, crowd behavior, event type, free hydration, alcohol availability, crowd mobility, venue design, length of event Reporting on patient presentation at in-event health service	Review Paper Discussion Paper Theoretical Discussion Conference Abstract Editorials

Papers were imported into Covidence (Veritas Health Innovation; Melbourne, Australia).^16^ The study selection process was carried out in three steps. First, two reviewers (NS and JR) reviewed the title and abstract of all articles, and a third reviewer (JC) resolved conflicts. Second, the same two reviewers (NS and JR) reviewed the full text of the articles to determine the eligibility of the study based on the inclusion and exclusion criteria outlined in Table 2, and a third reviewer (JC) resolved any disagreements in a blinded manner. Third, one reviewer (NS) extracted data manually from the included papers, a summary of which is outlined in Table 3 and Table 4. The second (JR), third (JC), and fourth reviewer (RW) split and crosschecked the data extracted.

**Table 3.** Summary of In-Event and External Health Services of Mass-Gathering Stadium/Arena Events.

Author(s), Year of Publication	Year of Event(s)	Cities/Countries	Event Type		MGE Attendees	In-Event Health Services		External to Event Health Services	
						Reported	Calculated	Reported	Calculated
Bhangu, et al, 2010^17^	2007-2008	Birmingham, UK	English Premier Football (21 matches)		Total: 816,658 Mean: 38,889	Raw: 78 Mean: 3.7 Median: 4 per game Mean PPR: 1/10,000	PPR: 0.9/10,000	Raw: 7 (9% of 78)	TTHR: 0.1/10,000
Chesshire & Gill, 1998^21^	1996-1997	London, UK	London Premiership Football (21 matches)		Average: 31,067	First Aid Raw: 122 Mean: 5.8 per match	PPR: 1.9/10,000	Ambulance Service: Raw: 1	TTHR: 0.0/10,000
						Health Care Professional Raw: 38 (among 122) Mean: 1.8 per match				ED: Raw: 6
						IR: 0.2/1000				
Crawford, et al, 2001^22^	1999-2000	Scotland, UK	Glasgow Celtic Football Club (26 matches)		Average: 51,271	Raw: 127 Mean: 4.9	PPR: 0.9/10,000	Ambulance Service: Raw: 20 (15.7% of 127)	TTHR: 0.2/10,000
								ED: Raw: 7 (5.5% of 127)		
De Lorenzo, et al, 1989^23^	1980-1986	New York, USA	Syracuse University Carrier Dome, indoor stadium games Football (42 matches), Basketball (133 games), Rock Concerts (25)		Football: Mean:36,335	Patient Volume: 11.4 PPR: 0.3/1000	PPR: 3.3/10,000	Ambulance Service: 10% of all events	TTHR: Football: 0.4/10,000 Basketball: 0.2/10,000 Concerts: 0.4/10,000
					Basketball: Mean: 19,627	Patient Volume: 5.0 PPR: 0.3/1000	PPR: 2.6/10,000	ED: Football: 10% Basketball: 5% Concerts: 5%		
					Concerts: Mean: 29,119	Patient Volume: 21.2 PPR: 0.9/1000	PPR: 9.8/10,000			
Elias, et al, 2020^24^	2019	Maputo City, Mozambique	Pope Francis’s visit 3^rd^ day: stadium event		Not reported	Raw: 112 (patients on 3^rd^ day)	–	Raw: 6	–
Erickson, et al, 1997^25^	1994	Chicago, USA	Rock Concert (n=5)		Total: 250,000 (approximate)	Raw: 308 Mean: 61.5 PPR: 1.2/1000	PPR: 12.3/10,000	Raw: 98 transported by paramedics to ED (32% of 308)	TTHR: 3.9/10,000
Hiltunen, et al, 2007^26^	2005	Helsinki, Finland	World Championship Games in Athletics (9 days)		Total: 479,000	Raw: 1586 PPR: 0.2/10,000	–	Raw: 25 TTHR: 0.5/10,000	–
Imbriaco, et al, 2020^27^	2019	Bologna, Italy	Union of European Football Associations’ Under-21 Championship (4 matches)		Total: 72655	Raw: 31 PPR: 0.4/1,000	PPR: 4.1/10,000	Raw: 3 TTHR: 0.1/ 1,000	TTHR: 1/10,000
Ishikawa, et al, 2007^28^	1996-2003	Tokyo, Japan	Baseball session in Meiji Jingu Baseball Stadium (67 games in 2003)		Total: 1,582,000 (During 2003)	Raw: 247 PPR: 3.7 per game	PPR: 1.6/10,000	Ambulance Service: Raw: 57 (1996-2003)	TTHR: 0.4/10,000
								ED: Raw: 10 (4.5% in 2003) Raw: 93 (During 1996-2003)		
Kao, et al, 2001^29^	1999	Taiwan	Summer Rock Concert Festival (2 days)		Total: 50,000	Raw: 28 PPR: 5.6/10,000	–	Raw: 1	TTHR: 0.2/10,000
Kman, et al, 2007^30^	2001-2005	Winston-Salem, North Carolina & Columbus, Ohio, USA	Division I College Football Games	Wake Forest Football (25 games)	Total: 687093	Mean PPR: 0.4/1000	Mean PPR: 4.5/10,000	Not reported	–
				Ohio State Football (22 games)	Total: 2296123	Mean PPR: 0.4/1000	Mean PPR: 4.3/10,000		
Leary, et al, 2017^31^	2002-2016	London, UK	English Football League (14 seasons)		Not reported	Raw: 981 PPR: (1.7-3.3) per 1,000 (Pre-implementation phase) PPR: (2.8-4.5) per 1,000 (Post-implementation phase)	Mean PPR: 2.9/10,000	Not reported	–
Lyons, et al, 2011^19^	2009	Birmingham, UK	Summer Cricket Sessions: (18 matches, 15 minor, 3 major)		Total: 183,387	First Aid Raw: 444	–	Raw: 7 (2% of 444)	TTHR: 0.4/10,000
						Health Care Professional Raw: 88				
						PPR: 24/10,000				
Milsten, et al, 2022^32^	2005-2016	Massachusetts, USA	Major League Baseball (MLB)		MLB1; Total: 8,995,742	Raw: 6,197 PPR: 0.2/10,000 (foul ball injury only)	–	TTHR: 0.0/10,000 (foul ball injury only)	–
						MLB2; Total: 9,668,463	Raw: 1,132 PPR: 0.1/10,000 (foul ball injury only)	–	TTHR: 0.0/10,000 (foul ball injury only)	–
						MLB3; Total: 16,398,399	Raw: 5,869 PPR: 0.2/10,000 (foul ball injury only)	–	TTHR: 0.4/10,000 (foul ball injury only)	–
Millán, et al, 2004^33^	1999	Seville, Spain	VII World Championships in Athletics		Total: 498,311	Raw: 1,338 PPR: 4.5/10,000	–	Raw: 35 (2.6% of 1.3)	TTHR: 0.7/10,000
Perron, et al, 2005^34^	1999-2003	Southeastern USA	Division I College Football: 20 matches		Range: 53,371 – 61,625 Mean: 56,327	Raw: 15-75 Mean: 36 PPR: (2.7 – 12.9) per 10,000 Mean PPR:6.3/10,000	–	Raw: 0 – 8 (range) Mean: 2.1	TTHR: 0.4/10,000
Shelton, et al, 1997^35^	1995	South Carolina, USA	University Football Season: 7 games		Total: 485,989 Mean: 69,427	Raw: 526 PPR: 1.1±0.4/1,000	PPR: 10.8/10,000	Raw: 19 (4% of 526)	TTHR: 0.4/10,000
Smith, et al, 2013^36^	2010	Manchester, UK	Soccer/Football Rugby Union (93 events)		Total: 6,061,890	Raw: 1,448	PPR: 2.3/10,000	Raw: 83 (6% of 1,448)	TTHR: 0.1/10,000
		Johannesburg, South Africa	Soccer/Football Rugby Union (66 events)		Total: 1,224,024	Raw: 266	PPR: 2.2/10,000	Raw: 19 (7% of 266)	TTHR: 0.2/10,000
Spaepen, et al, 2021^20^	2010-2019	Belgium	Football (41 matches)		Total: 1,630,549 Mean: 39,769	First Aid Raw: 626 Mean: 15.7	PPR: 3.8/10,000	Raw: 68 (52.7% of 626) TTHR: 0.4/1,000	TTHR: 4/10,000
						Health Care Professional Raw: 129 Mean: 3.2				
						PPR: 0.4/1,000				
Tajima, et al, 2020^37^	2019	Japan	Rugby World Cup 2019: 45 matches		Total: 1,704,443 Mean: 37,877	Raw: 449 PPR: 2.6/10,000	–	Raw: 38 TTHR: 0.2/10,000	–
Thompson, et al, 1991^38^	1988	Calgary, Canada	XV Winter Olympic Games		Total: 1.8 million	Raw: 796 PPR: 15/10,000	–	Ambulance Raw: 50 ED Raw: 1	TTHR: 0.3/10,000
Varon, et al, 2003^39^	1996-1997	Houston, Texas, USA	Indoor stadium complex: football, baseball, other sports, rodeo, concerts, trade shows (253 events)		Total: 3.3 million	Raw: 2762 PPR x/10,000 Baseball: 4.1 Football: 5.7 Rodeo: 9.2 Concerts: 2.9 Shows: 5.1	–	Ambulance Service: Raw: 50	TTHR: 0.2/10,000
								ED: Raw: 129		

Abbreviations: ED, Emergency Department; IR, Incidence Rate; MLB, Major League Baseball; MGE, Mass-Gathering Event; USA, United States of America; UK, United Kingdom; PPR, Patient Presentation Rate; TTHR, Transport to Hospital Rate.

**Table 4.** Factors Affecting Patient Presentation Rate and Transfer to Hospital Rate.

Article	Variables					
	Biomedical	Environmental	Psychosocial	Event Characteristics	Venue Characteristics	Health Support at the Stadium
Bhangu, et al, 2010^17^	Gender	Weather	Alcohol	Timing in relation to match:	Stadium infrastructure	-Crowd doctor -Ambulances -Major incident vehicle -First aiders
				-New injury/illness,	-Trips over broken pavement	
				-Exacerbation of pre-existing condition,	-Seating design	
				-Opportunistic presentation	-Entry turnstiles	
			Crowd behavior:	-Staff injury		
			-Fights			
			-Crushing			
			-Goal celebrations			
			-Hit by flying objects			
			Origin of injury:			
			-Crowd			
			-Stadium employee			
Chesshire & Gill, 2001^21^	–	–	–	–	–	-Crowd doctor -Ambulances -Major incident vehicle
Crawford, et al, 2001^22^	Age	–	Alcohol	Timing in relation to game	–	-3 doctors (ED, anesthetics, GP trained) -4 ambulance crews -Ambulance incident officer -Emergence support unit vehicle -2 paramedics+2 technicians -50-60 first aid workers
	Gender					
De Lorenzo, et al, 1989^23^	Age	Weather	Alcohol	Crowd size	- Moveable bleachers -Indoor/Outdoor	-First aid room (nurses, paramedics, EMTs, physician) -Teams of EMTs and paramedics deployed throughout the stadium -Ambulances for transport -Numbers of staff vary based on event
				Patient volume		
				Duration of the event: Total hour the stadium is opened for public		
	Gender					
Elias, et al, 2020^24^	Age	Weather	–	–	Open and uncovered stadium	Health post
	Gender					
	Diagnoses: Hypothermia					
Erickson, et al, 1997^25^	Age	–	Alcohol	Time of peak patient volume	Concerts sold out	-2 first aid medical facilities -Staffed by paramedics, nurses, senior emergency medicine residents, supervised by attending physician (emergency or toxicology trained) -Hours of operation (3pm-12pm) -Treatment (eg, IVT, wound care, O2) -Observation treatment time (mean 23,5mins) -Disposition (treated and released (32.5%), left against medical advice (35.5%), transported to hospital (32%); -Acuity: mild (67%), moderate (27%), severe (6%)
	Gender					
	Diagnosis: Trauma (minor/extreme), syncope, drug toxicity, gastro, weak/dizzy, head trauma, heat illness, dehydration, seizure		Drug			
Hiltunen, et al, 2007^26^	Age	Weather	–	–	Location of first aid stations	-Number of EMS personnel (9/day) -Hours of operation for the temporary rescue station (8a-11p) -Medical supervisor -Volunteer organization (for spectators and accredited persons) at stadium: 13 mobile first aid teams and 2 first aid facilities, 46 personnel (first aid education, healthcare professionals) -Emergency care provided (eg, O2, IVT, IVD, GTN, Blood gas) -Disposition: to ED by ambulance (n=14, 58%)
	Gender	-Temperature				
	Reason for call: Traumatic injury, medical, others	-Quality of air				
		-Rain				
Imbriaco, et al, 2020^27^	Age	Climate factors:	Alcohol	Availability of free water	–	-Stadium medical site -10 basic life support team with 4 Volunteer rescuers -2 ambulance with 1 emergency nurses and 1 emergency physicians -1 team (1 emergency nurse and 3 volunteer rescuers) -1 advance life support team (1 emergency nurse, 1 emergency physician and 1 rescuer) -2 bicycle teams
		-Mean temperature				
		-Heat index				
		-Humidity				
Ishikawa, et al, 2007^28^	Age	–	–	Time of occurrence -Before the start of the game -Near the end of the game		-First aid station -Ambulance
	Gender					
	Diagnosis					
Kao, et al, 2001^29^	Age	–	–	–	–	-Basic life support team -Ambulance
	Gender					
Kman, et al, 2007^30^	–	Weather: -Temperature	–	–	–	–
Leary, et al, 2017^31^	Age	–	–	–	–	-Crowd doctor, Physicians, nurses -St John Ambulance first aiders -London ambulance service
	Gender					
Lyons, et al, 2010^19^	Age	Weather	Alcohol	Crowd size	Design of stadium	-First aider, nurse, doctor, paramedic/EMT -Ambulance service -Blue light Ambulance
	Gender			Medical condition		
Milsten, et al, 2022^32^	Age	–	–	–	–	-First aid room -Ambulance service
	Gender					
Millán, et al, 2004^33^	Age	Temperature	–	Day of the event (opening/closing ceremony)	–	Number of clinics (spectators, athletes)
	Gender	Humidity		Crowd number		
	ICD-10 codes	Direction of wind				
	Attendee type (spectator, athlete, other)					
Perron, et al, 2005^34^	–	Heat index	–	–	–	–
Shelton, et al, 1997^35^	Age	–	–	–	Stadium capacity	Number of first aid stations,
	Gender				Tiered seating,	Location of first aid stations
	Type of complaint				Number of levels	Number of nurses, paramedics, doctors
Smith, et al, 2013^36^	Diagnosis severity (mild, moderate, severe)	–	–	Crowd attendance		Patient contact time (minutes)
Spaepen, et al, 2021^20^	Presenting problem	Temperature	–	Crowd attendance	Seating capacity,	-Number of first aid posts
	Patient disposition	Humidity		Type of game –Domestic,	Fenced perimeter to separate fans	-Type of health support (volunteers, emergency nurses, emergency physicians)
				-International friendly		
				-Qualifier		
Tajima, et al, 2020^37^	Age	Wet bulb globe temperature	–	Ticket sales numbers, Actual seats (crowd numbers)	Maximum capacity	Number of medical rooms,
	Gender					Medical room availability,
	Severity of illness (mild or severe)					Medical equipment/medication type availability,
	Nationality					Number of health staff,
						Health staff specialty type
						Time to transfer to hospital
Thompson, et al, 1991^38^	Acuity level (minor, moderate, serious, critical)					-Type of health support (first aid, nurses, physicians),
						Transport type (road ambulance, helicopter),
						Clinic type,
						Type of care (eg, sutures, intravenous)
Varon, et al, 2003^39^	Age		Alcohol availability	Event type	Seating capacity	Type of health staff (physicians, nurses, nurse practitioners, physician assistants, and emergency medical technicians)
	Gender		Drug use	Event-specific risks (eg, hit by baseball)		
	Attendee type			Attendee census		Encounter location
	45 diagnostic categories					Treatment type
						Disposition

Abbreviations: ED, Emergency Department; GP, General Practitioner; EMT, Emergency Medical Technician; EMS, Emergency Medical System; IVT, Intravenous Vitamin Therapy; IVD, In Vitro Diagnostics; GTN, Glyceryl Trinitrate; O2, Oxygen; ICD-10, International Classification of Diseases 10^th^ Revision.

### Data Collection and Data Synthesis

Charting the data (Stage 4) and collating, summarizing, and reporting the results (Stage 5) were undertaken, per Arskey and O’Mally.^15^ Information extracted from each article was recorded in two Microsoft 365 Word tables (Microsoft Corporation; Redmond, Washington USA). The first table included the author(s), year of publication, year of the MGE, country, MGE type, study population and sample size, in-event presentation, and hospital presentation. Two different ways were used to present in-event and hospital data. One method was to report the exact values found in the articles, such as raw values, mean values, PPR per 1,000 or 10,000, and TTHR per 1,000 or 10,000. The other method involved calculating the PPR and TTHR per 10,000, even when the original articles did not specify these measures in that format. The second table included factors affecting PPR and TTHR, grouped into six different domains; three (biomedical, environmental, psychosocial) from an earlier framework^2^ and three (event characteristics, venue characteristics, and health care characteristics) from further information elicited from this review. Data are reported using descriptive statistics.

## Results

**Figure 2.**
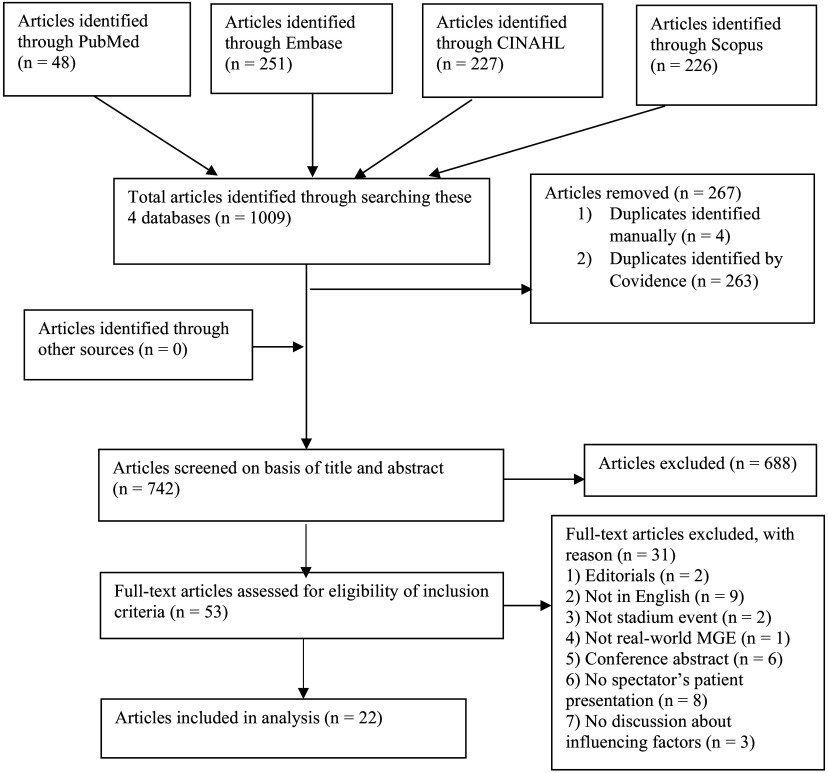
PRISMA Flow Diagram of Included Articles for this Scoping Review.

Out of 1,009 articles identified, 267 were duplicates and 688 were excluded at the title and abstract screening stage. The full text of 53 articles was reviewed, and after 31 were excluded, 22 articles that met criteria were included (Figure 2). Of the 22 articles, three (13.6%) were focused on concerts, one (4.6%) covered Pope Francis’s religious visit, and the remaining 18 (81.8%) were sports MGEs, including football, cricket, basketball, baseball, rugby, the Olympics, and the Athletics World Championships. Thirteen (59.1%) articles were MGEs in the United Kingdom (n = 6) and United States of America (n = 7). Table 317-39 provides a summary of the characteristics of the 22 included articles.

### In-Event Patient Presentation Rates

The PPR was reported in different ways. Some measured PPR as per 1,000 spectators (n = 7; 31.8%), while others presented it as per 10,000 spectators (n = 10; 45.5%). Some authors reported the total number of patients (n = 20; 90.9%), the mean number of patients (n = 6; 27.3%), or the PPR per game (n = 1; 4.6%). When the PPR for each article was adjusted to 10,000 by the authors of this review, the PPR varied from one to 24 per 10,000, and the median PPR was 3.8 per 10,000 spectators. The highest reported PPR was noted to occur during the 2009 Summer Cricket Season in the United Kingdom (24 per 10,000).

### Transfer to Hospital Rates

During MGEs, patients may require an ambulance transfer to a hospital’s ED due to needing higher levels of care. Whilst some articles measured the TTHR per 1,000 spectators (n = 2; 9.1%) or 10,000 spectators (n = 3; 13.6%), most articles reported the total number (n = 15; 68.2%) and/or the percentage (n = 10; 45.5%) of spectators who received in-event health care and were transported to the hospital from the MGE. When the TTHR for each article was adjusted to 10,000 by the authors of this review, the TTHR varied from 0.01 to four per 10,000, and the median TTHR was 0.35 per 10,000 spectators. The highest TTHR was during the football season in Belgium, which was four per 10,000 spectators.

### Factors Reported for PPR and TTHR

Biomedical, environmental, and psychosocial factors that may have contributed to PPR and TTHR that were reported by authors are summarized in Table 4.^17-39^

Age and gender were common biomedical factors reported, noted in 15 (68.2%) articles. Male (n = 10) spectators were more likely to be injured than females (n = 5). The severity of illness was also a biomedical factor reported in some articles (n = 7; 31.8%). The weather was identified as an important environmental factor in the majority of articles (n = 12; 54.6%). Weather factors reported included temperature (minimum and maximum), such as an increase in daily maximum temperatures; heat index (the perceived temperature influenced by both temperature and humidity); air quality (classified as good, fair, or poor, depending on the presence of dust, gas, mist, odor, or smoke in crowded places); rainfall; humidity (average); and wind direction (which helps measure weather patterns). The first two factors, temperature and heat index, were the most commonly reported. A statistical correlation was found between the game-time heat index and the volume of patients; however, cold weather also affected spectators’ health. Psychosocial factors reported included alcohol consumption (n = 7; 31.8%), drug use (n = 2; 9.1%), and crowd behavior (n = 1; 4.6%). Of these, alcohol consumption was a factor that considerably influenced PPR and TTR.

In addition to biomedical, environmental, and psychosocial factors, other categories of factors were identified, which authors categorized as event-specific characteristics, venue characteristics, and health support at the venue as these varied from event to event. Event characteristics were reported such as crowd size (n = 8; 36.4%), the timing of the event (n = 4; 18.2%), availability of drinking water (n = 1; 4.6%), and day of the event (opening/closing ceremony; n = 1; 4.6%). Venue characteristics included venue infrastructure, which was mostly permanent in stadiums or arenas. Reported issues with venue infrastructure pertained to broken pavement, entry turnstiles, uneven flooring, broken handrails, seating design, number of levels, indoor or outdoor setting, fenced perimeter, maximum capacity (n = 4; 18.2%), and the location of the first aid station (n = 1; 4.5%). Health support characteristics reported included the number of first aid stations (n = 10; 45.5%) and the presence of health care providers including doctors, nurses, paramedics/Emergency Medical Technicians (EMTs), first aiders, and Basic Life Support teams (n = 14; 63.6%). Additionally, having enough medical equipment (n = 3; 13.6%), ambulances and trained ambulance crew (n = 12; 54.6%), treatment type (n = 6; 27.3%), and shorter transfer times to the hospital (n = 1; 4.6%) were factors that were reported to reduce the vulnerability of patients during MGEs.

## Discussion

**Figure 3.**
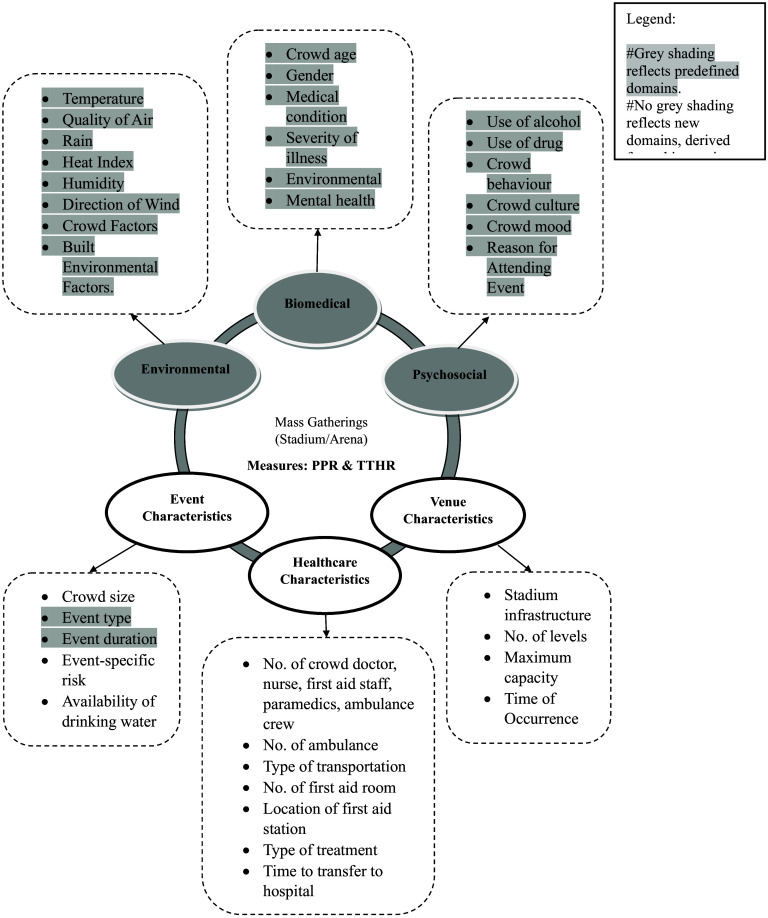
Proposed Framework for Stadium/Arena Event Mass Gatherings.

This review identified three key findings regarding presentations to health services at MGEs. First, three additional domains were identified to extend upon Arbon’s earlier framework^2^ for MGEs that are specific to stadiums/arenas. Second, PPR and TTHR are variably reported and vary considerably. Third, factors influencing PPR and TTHR that are specific to stadiums/arenas are articulated according to the six domains outlined. With the emerging evidence on stadium and arena MGEs identified and reported on in this scoping review, along with the three traditional biomedical, environmental, and psychosocial domains identified by Arbon,^2^ authors encourage others planning future research or planning future MGEs being held in stadiums or arenas to consider three additional domains: event characteristics, venue characteristics, and health support characteristics. As depicted in Figure 3, the expanded framework accounts for the consideration of certain event characteristics, venue characteristics, and availability and type of health support at the MGE, which can vary depending on the event.

This review highlighted that patient-level data are variably reported in articles about stadiums and arenas. Important metrics such as PPR and TTHR are reported in different units^2^ ranging from per 100 to per 10,000. While there have been several articles on PPR and TTHR at MGEs, the majority haven’t been on stadium or arena MGEs.^12^ Standardizing and systematizing the measurements of health outcomes associated with MGEs will enhance the accuracy and reliability of reported data, contributing to the overall quality of epidemiological information.^12^ A consistent measure of stadium safety may be the occurrence of major incidents.^17^ To gain a better understanding of health care needs at stadiums and arenas, it must be ensured that the same variables and units of measurement are consistently reported, an issue and recommendation noted by others.^18^

This article presents a framework of six domains that can be viewed as determinants, similar to Arbon’s prediction model.^11^ Interestingly, there is a lack of comprehensive studies on stadium/arena MGEs that illustrate the relationship between these factors. Most articles simply present statistical data in terms of frequencies and percentages. For instance, crowd numbers are a common factor that significantly influences the PPR and TTHR.^19,20^ However, other crucial factors need to be considered to understand their impact on PPR and TTHR. Nevertheless, predicting the PPR and TTHR is critical for well-organized MGEs. A comprehensive understanding of PPR and TTHR requires exploring their associations with all the different factors related to stadium and arena MGEs. The importance of the factors will be assessed in future research, using statistical models against patient level data from multiple stadium and arena events.

## Study Limitations

Despite the existing research, gaps remain in understanding health care provision in stadium and arena settings and in applying findings to different types of events and global contexts. The use of different measuring tools makes it challenging to summarize information about PPR and TTHR. To enhance the provision of optimal health care services at mass-gathering stadium/arena events, all major factors contributing to PPR and TTHR need to be taken into consideration. The factors have been derived from the reported variables within the reviewed papers; these variables have not been mathematically validated in these papers. More advanced statistical predictive models are one approach that can be used to predict PPR and TTHR, taking into account all major determinants. Addressing these challenges can help stakeholders better prepare for and support the health and safety of attendees, making MGEs enjoyable and secure for participants.

## Conclusion

This scoping review offers a comprehensive overview of the factors that influence health care utilization and outcomes during stadium and arena MGEs. The review emphasizes the intricate interplay of biomedical, environmental, psychosocial, event-specific, venue-related, and health care support factors. Even though the proposed conceptual model is specifically for stadium and arena MGEs, the model has relevance and adaptability to the non-stadium MGEs. It highlights the need for personalized and forward-thinking health care planning and resource allocation to keep participants safe and healthy at future MGEs. By gaining a deeper understanding of these influencing factors, it is possible to improve the effectiveness of health care strategies for MGEs and to ensure the well-being of all participants.

## Supporting information

Sultana et al. supplementary material Sultana et al. supplementary material

## Supplementary Material

To view supplementary material for this article, please visit https://doi.org/10.1017/S1049023X25000287
