# Supplementary material for: Factors Influencing Patient Presentation and Transfer to Hospital Rates During Mass-Gathering Stadium Events: A Scoping Review
Source: Prehosp Disaster Med. 2025 Apr 10;40(2):101–13. doi: 10.1017/S1049023X25000287 (PMC12018010; doi:10.1017/S1049023X25000287)
Supplement: Sultana et al. supplementary material [file S1049023X25000287sup001.docx]

**SUPPLEMENTARY MATERIAL**

**Results from four database searches**

**PUBMED Search:**

| **Search Number** | **Query** | **Search Details** | **Results** |
| --- | --- | --- | --- |
| 33 | #30 AND #31 AND #32 | ("mass gatherings"[MeSH Terms] OR "crowding"[MeSH Terms] OR "anniversaries and special events"[MeSH Terms] OR "large event*"[Text Word] OR "major event*"[Text Word] OR "mass event*"[Text Word] OR "event planning*"[Text Word]) AND ("track and field"[MeSH Terms] OR "stadium*"[Text Word] OR "arena*"[Text Word] OR "ground*"[Text Word] OR "filed*"[Text Word] OR "colosseum*"[Text Word]) AND ("first aid"[MeSH Terms] OR "ambulances"[MeSH Terms] OR "emergency medical services"[MeSH Terms] OR "health personnel"[MeSH Terms] OR "emergency treatment"[MeSH Terms] OR "emergency medical technicians"[MeSH Terms] OR "nurses"[MeSH Terms] OR "physicians"[MeSH Terms] OR "medical staff"[MeSH Terms] OR "sports medicine"[MeSH Terms] OR "doctor*"[Text Word] OR "healthcare*"[Text Word] OR "patient presentations*"[Text Word] OR "transport to hospital*"[Text Word] OR "paramedic*"[Text Word] OR "medical care*"[Text Word]) | 48 |
| 32 | #14 OR #15 OR #16 OR #17 OR #18 OR #19 OR #20 OR #21 OR #22 OR #23 OR #24 OR #25 OR #26 OR #27 OR #28 OR #29 | "first aid"[MeSH Terms] OR "ambulances"[MeSH Terms] OR "emergency medical services"[MeSH Terms] OR "health personnel"[MeSH Terms] OR "emergency treatment"[MeSH Terms] OR "emergency medical technicians"[MeSH Terms] OR "nurses"[MeSH Terms] OR "physicians"[MeSH Terms] OR "medical staff"[MeSH Terms] OR "sports medicine"[MeSH Terms] OR "doctor*"[Text Word] OR "healthcare*"[Text Word] OR "patient presentations*"[Text Word] OR "transport to hospital*"[Text Word] OR "paramedic*"[Text Word] OR "medical care*"[Text Word] | 1,334,237 |
| 31 | #8 OR #9 OR #10 OR #11 OR #12 OR #13 | "track and field"[MeSH Terms] OR "stadium*"[Text Word] OR "arena*"[Text Word] OR "ground*"[Text Word] OR "filed*"[Text Word] OR "colosseum*"[Text Word] | 245,698 |
| 30 | #1 OR #2 OR #3 OR #4 OR #5 OR #6 OR #7 | "mass gatherings"[MeSH Terms] OR "crowding"[MeSH Terms] OR "anniversaries and special events"[MeSH Terms] OR "large event*"[Text Word] OR "major event*"[Text Word] OR "mass event*"[Text Word] OR "event planning*"[Text Word] | 14,278 |
| 29 | "Medical care*"[tw] | "medical care*"[Text Word] | 61,061 |
| 28 | Paramedic*[tw] | "paramedic*"[Text Word] | 9,921 |
| 27 | "Transport to hospital*"[tw] | "transport to hospital*"[Text Word] | 212 |
| 26 | "Patient presentations*"[tw] | "patient presentations*"[Text Word] | 551 |
| 25 | Healthcare*[tw] | "healthcare*"[Text Word] | 381,320 |
| 24 | Doctor*[tw] | "doctor*"[Text Word] | 149,282 |
| 23 | Sports Medicine [Mesh] | "sports medicine"[MeSH Terms] | 11,522 |
| 22 | Medical staff [Mesh] | "medical staff"[MeSH Terms] | 28,775 |
| 21 | Physicians [Mesh] | "physicians"[MeSH Terms] | 173,146 |
| 20 | Nurses [Mesh] | "nurses"[MeSH Terms] | 97,287 |
| 19 | Emergency Medical Technicians [Mesh] | "emergency medical technicians"[MeSH Terms] | 6,000 |
| 18 | Emergency treatment [Mesh] | "emergency treatment"[MeSH Terms] | 133,632 |
| 17 | Health Personnel [Mesh] | "health personnel"[MeSH Terms] | 601,845 |
| 16 | Emergency medical services [Mesh] | "emergency medical services"[MeSH Terms] | 166,258 |
| 15 | Ambulances [Mesh] | "ambulances"[MeSH Terms] | 9,809 |
| 14 | First aid [Mesh] | "first aid"[MeSH Terms] | 8,077 |
| 13 | Colosseum*[tw] | "colosseum*"[Text Word] | 9 |
| 12 | Filed*[tw] | "filed*"[Text Word] | 4,744 |
| 11 | Ground*[tw] | "ground*"[Text Word] | 219,645 |
| 10 | Arena*[tw] | "arena*"[Text Word] | 19,472 |
| 9 | Stadium*[tw] | "stadium*"[Text Word] | 1,299 |
| 8 | Track and field [Mesh] | "track and field"[MeSH Terms] | 1,048 |
| 7 | "Event planning*"[tw] | "event planning*"[Text Word] | 94 |
| 6 | "Mass event*"[tw] | "mass event*"[Text Word] | 103 |
| 5 | "Major event*"[tw] | "major event*"[Text Word] | 2,596 |
| 4 | "Large event*"[tw] | "large event*"[Text Word] | 240 |
| 3 | Anniversaries and special events [Mesh] | "anniversaries and special events"[MeSH Terms] | 7,492 |
| 2 | Crowding [Mesh] | "crowding"[MeSH Terms] | 3,862 |
| 1 | Mass gathering [Mesh] | "mass gatherings"[MeSH Terms] | 117 |

**Embase Search:**

| **No.** | **Query** | **Results** |
| --- | --- | --- |
| 33 | #8 AND #15 AND #32 | 251 |
| 32 | #16 OR #17 OR #18 OR #19 OR #20 OR #21 OR #22 OR #23 OR #24 OR #25 OR #26 OR #27 OR #28 OR #29 OR #30 OR #31 | 3,336,028 |
| 31 | 'medical care' | 248,758 |
| 30 | paramedic* | 41,785 |
| 29 | 'transport to hospital' | 322 |
| 28 | 'patient presentation*' | 2,996 |
| 27 | healthcare | 1,095,508 |
| 26 | doctor* | 444,684 |
| 25 | 'sports medicine' | 115,898 |
| 24 | 'medical staff' | 55,246 |
| 23 | physician* | 1,054,322 |
| 22 | nurse* | 598,493 |
| 21 | 'emergency medical technician*' | 1,819 |
| 20 | 'emergency treatment' | 22,776 |
| 19 | 'health care personnel' | 229,257 |
| 18 | 'emergency health service' | 116,002 |
| 17 | ambulance* | 28,737 |
| 16 | 'first aid' | 16,332 |
| 15 | #9 OR #10 OR #11 OR #12 OR #13 OR #14 | 1,633,898 |
| 14 | colosseum* | 96 |
| 13 | field* | 1,345,826 |
| 12 | ground* | 269,933 |
| 11 | arena* | 40,599 |
| 10 | stadium* | 10,377 |
| 9 | track and field' | 1,565 |
| 8 | #1 OR #2 OR #3 OR #4 OR #5 OR #6 OR #7 | 14,355 |
| 7 | 'event plan*' | 164 |
| 6 | 'mass event*' | 170 |
| 5 | 'major event*' | 3,786 |
| 4 | 'large event*' | 244 |
| 3 | 'anniversaries and special events' | 11 |
| 2 | 'crowding (area)' | 8,811 |
| 1 | 'mass gathering*' | 1,459 |

**CINAHL Search:**

| **Search number** | **Query** | **Results** |
| --- | --- | --- |
| 33 | #30 AND #31 AND #32 | 227 |
| 32 | #14 OR #15 OR #16 OR #17 OR #18 OR #19 OR #20 OR #21 OR #22 OR #23 OR #24 OR #25 OR #26 OR #27 OR #28 OR #29 | 1,600,609 |
| 31 | #8 OR #9 OR #10 OR #11 OR #12 OR #13 | 214,473 |
| 30 | #1 OR #2 OR #3 OR #4 OR #5 OR #6 OR #7 | 28,702 |
| 29 | "Medical care*" | 28,980 |
| 28 | Paramedic* | 101 |
| 27 | "Transport to hospital*" | 212 |
| 26 | "Patient presentation*" | 785 |
| 25 | Healthcare* | 771,489 |
| 24 | Doctor* | 112,496 |
| 23 | "Sports Medicine" | 18,651 |
| 22 | "Medical staff" | 11,223 |
| 21 | Physician* | 282,211 |
| 20 | Nurse* | 597,832 |
| 19 | "Emergency Medical Technicians" | 13,452 |
| 18 | "Emergency treatment" | 41,231 |
| 17 | "Health Personnel" | 117,478 |
| 16 | "Emergency medical services" | 32,650 |
| 15 | "Ambulance*" | 10,195 |
| 14 | "First aid" | 4,497 |
| 13 | Colosseum* | 9 |
| 12 | Filed* | 152,169 |
| 11 | Ground* | 53,092 |
| 10 | Arena* | 7,636 |
| 9 | Stadium* | 606 |
| 8 | "Track and field" | 7,314 |
| 7 | Crowd* | 7469 |
| 6 | "Mass event*" | 32 |
| 5 | "Major event*" | 486 |
| 4 | "Large event*" | 30 |
| 3 | "Anniversaries and special events*" | 20,171 |
| 2 | Crowding | 3,704 |
| 1 | "Mass gathering*" | 770 |

**Scopus Search:**

| **Combine Queries** | **Searched for** | **Results** |
| --- | --- | --- |
| 10 | Crowding OR "Mass gathering" OR "Anniversaries and special events" OR ("large event" OR "large events") OR ("major event" OR "major events") OR ("Mass event " OR "Mass events") OR (Event planning ) | 260 |
| 18 | "Track and Field" OR Stadium* OR Arena* OR Ground* OR Field* OR Colosseum* | 2,292,135 |
| 37 | "First Aid" OR Ambulance* OR "Emergency medical services" OR "Health Personnel" OR "Emergency treatment" OR "Emergency Medical Technician*" OR Nurse* OR Physician* OR "Medical staff" OR "Sports Medicine" OR Doctor* OR Healthcare OR "Patient presentation*" OR "Transport to hospital" OR Paramedic OR "Medical care" | 7,278,946 |
| 38 | (Crowding OR "Mass gathering" OR "Anniversaries and special events" OR ("large event" OR "large events") OR ("major event" OR "major events") OR ("Mass event " OR "Mass events") OR (Event planning )) AND ("Track and Field" OR Stadium* OR Arena* OR Ground* OR Field* OR Colosseum*) AND ("First Aid" OR Ambulance* OR "Emergency medical services" OR "Health Personnel" OR "Emergency treatment" OR "Emergency Medical Technician*" OR Nurse* OR Physician* OR "Medical staff" OR "Sports Medicine" OR Doctor* OR Healthcare OR "Patient presentation*" OR "Transport to hospital" OR Paramedic OR "Medical care") | 49,961 |

**Preferred Reporting Items for Systematic reviews and Meta-Analyses extension for Scoping Reviews (PRISMA-ScR) Checklist**

| **SECTION** | **ITEM** | **PRISMA-ScR CHECKLIST ITEM** | **REPORTED ON PAGE #** |
| --- | --- | --- | --- |
| **TITLE** | | | |
| Title | 1 | Identify the report as a scoping review. | Titel; Page 2 |
| **ABSTRACT** | | | |
| Structured summary | 2 | Provide a structured summary that includes (as applicable): background, objectives, eligibility criteria, sources of evidence, charting methods, results, and conclusions that relate to the review questions and objectives. | Abstract; Page 2 |
| **INTRODUCTION** | | | |
| Rationale | 3 | Describe the rationale for the review in the context of what is already known. Explain why the review questions/objectives lend themselves to a scoping review approach. | Introduction, paragraph 1 & 2; Page 4 |
| Objectives | 4 | Provide an explicit statement of the questions and objectives being addressed with reference to their key elements (e.g., population or participants, concepts, and context) or other relevant key elements used to conceptualize the review questions and/or objectives. | Introduction, paragraph 3; Page 5 |
| **METHODS** | | | |
| Protocol and registration | 5 | Indicate whether a review protocol exists; state if and where it can be accessed (e.g., a Web address); and if available, provide registration information, including the registration number. | Method: Design; Page 5 |
| Eligibility criteria | 6 | Specify characteristics of the sources of evidence used as eligibility criteria (e.g., years considered, language, and publication status), and provide a rationale. | Table 2; Page 22 |
| Information sources* | 7 | Describe all information sources in the search (e.g., databases with dates of coverage and contact with authors to identify additional sources), as well as the date the most recent search was executed. | Method: Search strategy; Page 5 |
| Search | 8 | Present the full electronic search strategy for at least 1 database, including any limits used, such that it could be repeated. | Supplementary material; Page 35-39 |
| Selection of sources of evidence† | 9 | State the process for selecting sources of evidence (i.e., screening and eligibility) included in the scoping review. | Method: Search strategy; Page 5, Table 2; Page 22 |
| Data charting process‡ | 10 | Describe the methods of charting data from the included sources of evidence (e.g., calibrated forms or forms that have been tested by the team before their use, and whether data charting was done independently or in duplicate) and any processes for obtaining and confirming data from investigators. | Method: data collection and data synthesis; Page 6/7 |
| Data items | 11 | List and define all variables for which data were sought and any assumptions and simplifications made. | Table 3; Page 23  Table 4; Page 28 |
| Critical appraisal of individual sources of evidence§ | 12 | If done, provide a rationale for conducting a critical appraisal of included sources of evidence; describe the methods used and how this information was used in any data synthesis (if appropriate). | NA |
| Synthesis of results | 13 | Describe the methods of handling and summarizing the data that were charted. | Method: data collection and data synthesis; Page 6/7 |
| **RESULTS** | | | |
| Selection of sources of evidence | 14 | Give numbers of sources of evidence screened, assessed for eligibility, and included in the review, with reasons for exclusions at each stage, ideally using a flow diagram. | Figure 2; Page 19 |
| Characteristics of sources of evidence | 15 | For each source of evidence, present characteristics for which data were charted and provide the citations. | Table 3; Page 23  Table 4; Page 28 |
| Critical appraisal within sources of evidence | 16 | If done, present data on critical appraisal of included sources of evidence (see item 12). | NA |
| Results of individual sources of evidence | 17 | For each included source of evidence, present the relevant data that were charted that relate to the review questions and objectives. | Table 3; Page 23  Table 4; Page 28 |
| Synthesis of results | 18 | Summarize and/or present the charting results as they relate to the review questions and objectives. | Results, Paragraph 1, 2 & 3; Page 7 & 8 |
| **DISCUSSION** | | | |
| Summary of evidence | 19 | Summarize the main results (including an overview of concepts, themes, and types of evidence available), link to the review questions and objectives, and consider the relevance to key groups. | Discussion; Page 9/10 |
| Limitations | 20 | Discuss the limitations of the scoping review process. | Study limitations; Page 11 |
| Conclusions | 21 | Provide a general interpretation of the results with respect to the review questions and objectives, as well as potential implications and/or next steps. | Conclusion; Page 11 |
| **FUNDING** | | | |
| Funding | 22 | Describe sources of funding for the included sources of evidence, as well as sources of funding for the scoping review. Describe the role of the funders of the scoping review. | NA |
